# Permafrost-related hazard, vulnerability and risk estimates for cultural heritage and modern buildings in Svalbard

**DOI:** 10.1038/s41597-026-07568-7

**Published:** 2026-06-06

**Authors:** Ionut Cristi Nicu, Line Rouyet, Lena Rubensdotter, Anatoly Sinitsyn, Lotte Wendt, Hanne H. Christiansen, Lise Loktu

**Affiliations:** 1 High North Department, Norwegian Institute for Cultural Heritage Research (NIKU), Fram Centre, N-9296 Tromsø, Norway; 2 NORCE Research AS, Siva Innovasjonssenter, P.O. Box 6434, 9294 Tromsø, Norway; 3 Geohazard and Earth Observation Section, Geological Survey of Norway (NGU), P.O. Box 6315 Torgarden, 7491 Trondheim, Norway; 4 Arctic Geology Department, The University Centre in Svalbard (UNIS), P.O. Box 156, 9171 Longyearbyen, Norway; 5 SINTEF AS, S.P. Andersens veg 3, N-7031 Trondheim, Norway; 6 Department of Environmental Science, Aarhus University, Frederiksborgvej 399, 4000 Roskilde, Denmark; 7 Arctic Geophysics Department, The University Centre in Svalbard (UNIS), P.O. Box 156, 9171 Longyearbyen, Norway

## Abstract

With the ongoing climate changes, the Arctic is experiencing large changes, which have the potential to negatively influence natural environments, human settlements, and cultural heritage. The permanently frozen ground (permafrost) and the seasonally thawing and freezing active layer are sensitive to changes in temperature and precipitation patterns. With permafrost degradation and active layer thickening, Arctic cultural heritage and modern buildings can be increasingly affected by permafrost-related hazards. Mitigating these hazards requires implementation of tools at the local to regional scale. This paper presents a spatial dataset of permafrost-related hazards in Svalbard, integrating three geospatial indicators documenting (1) geomorphology-based hazard susceptibility, (2) Interferometric Synthetic Aperture Radar (InSAR) ground movement, and (3) coastal erosion hazard susceptibility based on the distance to the coastlines. The resulting hazard indicator is combined with vulnerability indicators to provide risk estimates for cultural heritage sites and modern buildings in and around Longyearbyen and Ny-Ålesund. The dataset includes the products from all steps, from the initial spatial geodata to the hazard/vulnerability indicators and risk estimates.

## Background & Summary

Permafrost covers approximately one quarter of the Northern Hemisphere’s land area and is a fundamental component of Arctic environments, ecosystems, and societies^1,2^. In recent decades, the permafrost in the Arctic region has undergone accelerated warming, leading to an increase in ground temperatures^3,4^. These changes directly affect terrain stability, surface hydrology, and geomorphological processes, resulting in widespread thaw subsidence^5–7^, solifluction^8–10^, retrogressive thaw slumps^11–14^, thermo-erosion gullying^15–17^, and erosion in coastal^18–20^ and riverine systems^21–23^. Furthermore, climate projections indicate that permafrost degradation and associated hazards will intensify throughout the 21^st^ century under ongoing global warming^24–26^.

Svalbard represents a particularly critical environment in this context, as one of the fastest-warming regions worldwide, with recent analyses showing air temperature increases exceeding four times the global mean^3,27,28^. Consequently, the frequency and intensity of cryospheric hazards associated with permafrost changes and thawing is likely to increase. These transformations may have direct societal implications. Svalbard’s communities and infrastructure, including but not limited to modern buildings (MB) and linear infrastructure (e.g., roads, pipelines), are predominantly located in low-lying permafrost landscapes close to fjords and coastal areas^29^. Cultural heritage sites (CH), encompassing archaeological remains of early human activity on Svalbard, including European whaling, Russian and Norwegian fur trapping, and more recent historical remains related to industrial heritage from coal mining and scientific explorations, are similarly exposed to permafrost degradation and associated hazards^30–33^.

In the context of Svalbard, where CH represents a unique global heritage as well as an integral component of both scientific research and legal obligations under heritage protection frameworks, the urgency of systematic monitoring and risk assessment is particularly pronounced^10,15,18,33–35^. Nevertheless, despite considerable scientific advances in the field of permafrost modelling, permafrost field monitoring and large-scale permafrost hazard assessments^20,29,36–38^, significant knowledge gaps persist in consistent risk assessment at the local to regional scale. The resolution of Pan-Arctic permafrost hazard models and risk assessments is often inadequate for detailed local decision-making and policy frameworks in intensively used settlements, such as Longyearbyen and Ny-Ålesund.

In Svalbard, several past studies have focused on geomorphological mapping^39–43^, InSAR ground movement mapping^44,45^, and coastal erosion affecting CH^18,35^. However, studies combining these different components towards a more comprehensive hazard and risk assessment have been so far lacking.

To address this gap, we developed a spatially explicit dataset designed for the assessment of permafrost-related risks for CH and MB in Longyearbyen and Ny-Ålesund, Svalbard. We integrated three complementary indicators: (1) geomorphology-based hazard susceptibility, (2) Interferometric Synthetic Aperture Radar (InSAR) ground movement, and (3) coastal erosion hazard susceptibility based on the distance to the coastlines. The resulting hazard indicator is combined with CH and MB vulnerability indicators to provide risk estimates. The results consist in a comprehensive inventory of qualitative scores for 639 (Longyearbyen) and 90 (Ny-Ålesund) CH, as well as 1028 (Longyearbyen) and 128 (Ny-Ålesund) MB. The dataset is meant to facilitate further prioritisation of risk mitigation and adaption measures, proposing a combined analysis of permafrost-related hazards and human-valued assets. This new data resource supports a wide range of applications, including geoscientific modelling of permafrost-landscape interactions^46,47^, CH protection planning^48,49^ and engineering-oriented infrastructure assessments^50,51^. Risk assessment of CH sites and MB needs to be able to integrate information on both sudden events (e.g., rockfalls) and slow processes (e.g., coastal erosion, ground movement due to permafrost degradation) to adequately support prioritisation of future risk management strategies^52^.

The present work is intended to be of direct relevance to local stakeholders and policymakers, cultural heritage managers and conservators, as well as Arctic researchers, climate and permafrost scientists, working at the interface between science and society. For CH researchers, such as historians and archaeologists, it aids in prioritising historical documentation. For the tourist industry, it might contribute to planning sustainable tourism strategies^53,54^. For CH authorities and managers (e.g., the Governor of Svalbard (Sysselmesteren), the Norwegian Directorate for Cultural Heritage (Riksantikvaren), Svalbard Museum), and the MB owners and managers (e.g., The Longyearbyen Local Council (Lokalstyre), The Norwegian Directorate of Public Construction and Property (Statsbygg), Store Norske, Kings Bay), the dataset will provide them with a list of objects with assigned hazard, vulnerability and risk scores to support informed decisions on resource allocation, maintenance planning, prioritisation and adaptation strategies^55–58^. For local stakeholders and policymakers, the dataset is meant to serve as a supporting tool to enhance communication on permafrost-related hazard and inform risk adaptation and mitigation strategies^37,59,60^.

Beyond Svalbard, this new dataset contributes to pan-Arctic efforts, as well as initiatives in other cold regions at lower latitudes (such as mountain regions), to assess and mitigate the cascading impacts of permafrost degradation on both CH^30^ and MB^61^. This initiative serves to demonstrate how open data initiatives can be leveraged to foster cross-disciplinary collaboration, align with international goals for cultural and environmental sustainability and climate adaptation in regions undergoing rapid permafrost transformations^62–66^.

### Study area

**Fig. 1:**
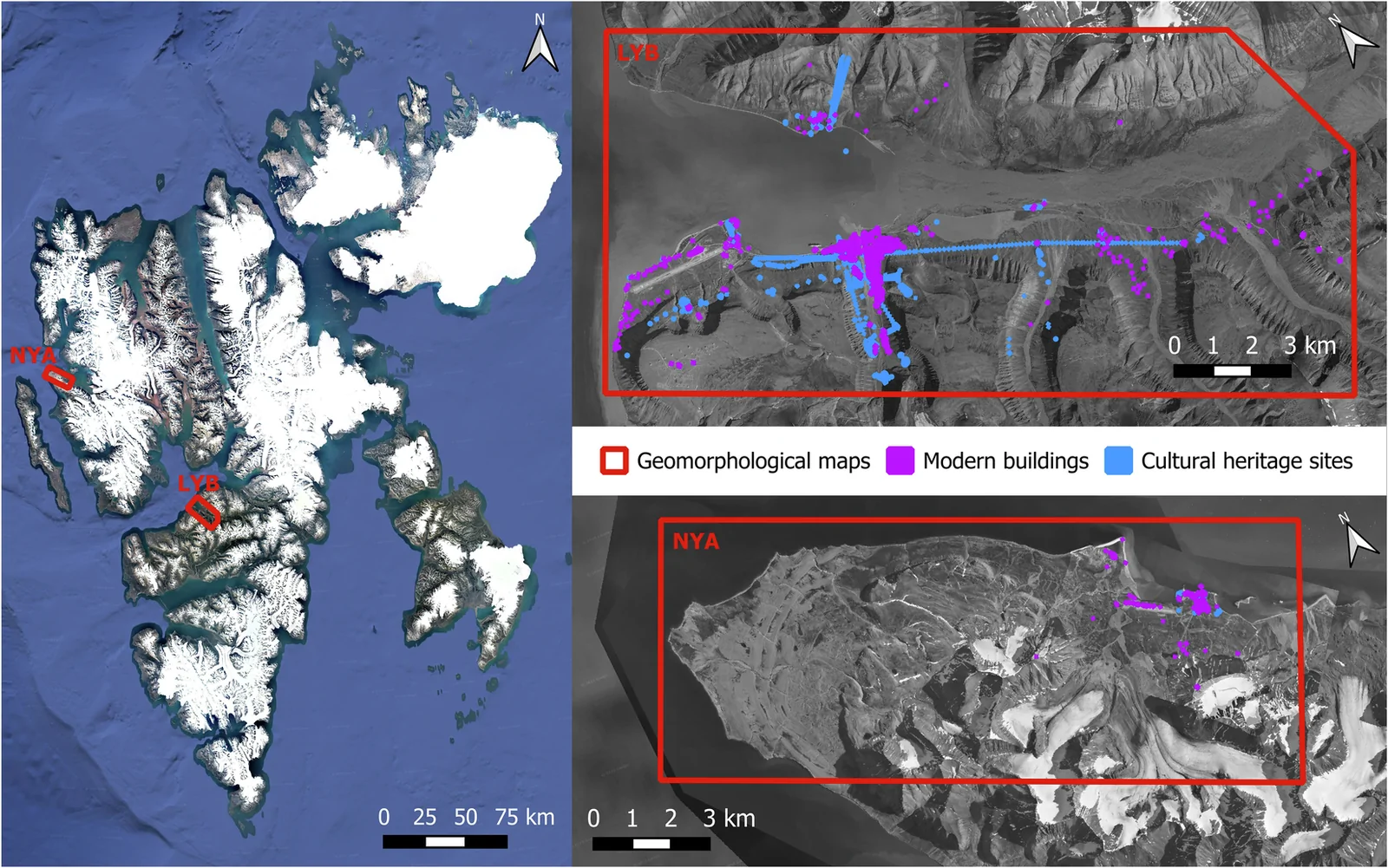
Location map of the two study areas (LYB: Longyearbyen and NYA: Ny-Ålesund) in the Svalbard Archipelago. Backgrounds: Orthoimages from the Google Satellite Web Map Service and Svalbard orthomosaics from the Norwegian Polar Institute^70^.

The Svalbard Archipelago has an area of approximately 61,020 km^2^ and is situated halfway between the northern coast of Norway and the geographical North Pole. Spitsbergen is the largest island in Svalbard and the only one with a permanent population^67^ (Fig. 1). The focus in this paper is on the two most important Norwegian settlements Longyearbyen^68^ and Ny-Ålesund^51^. Permafrost is widespread in Svalbard outside the glaciers, and the warmest this far north in the Arctic^4^.

## Methods

**Fig. 2:**
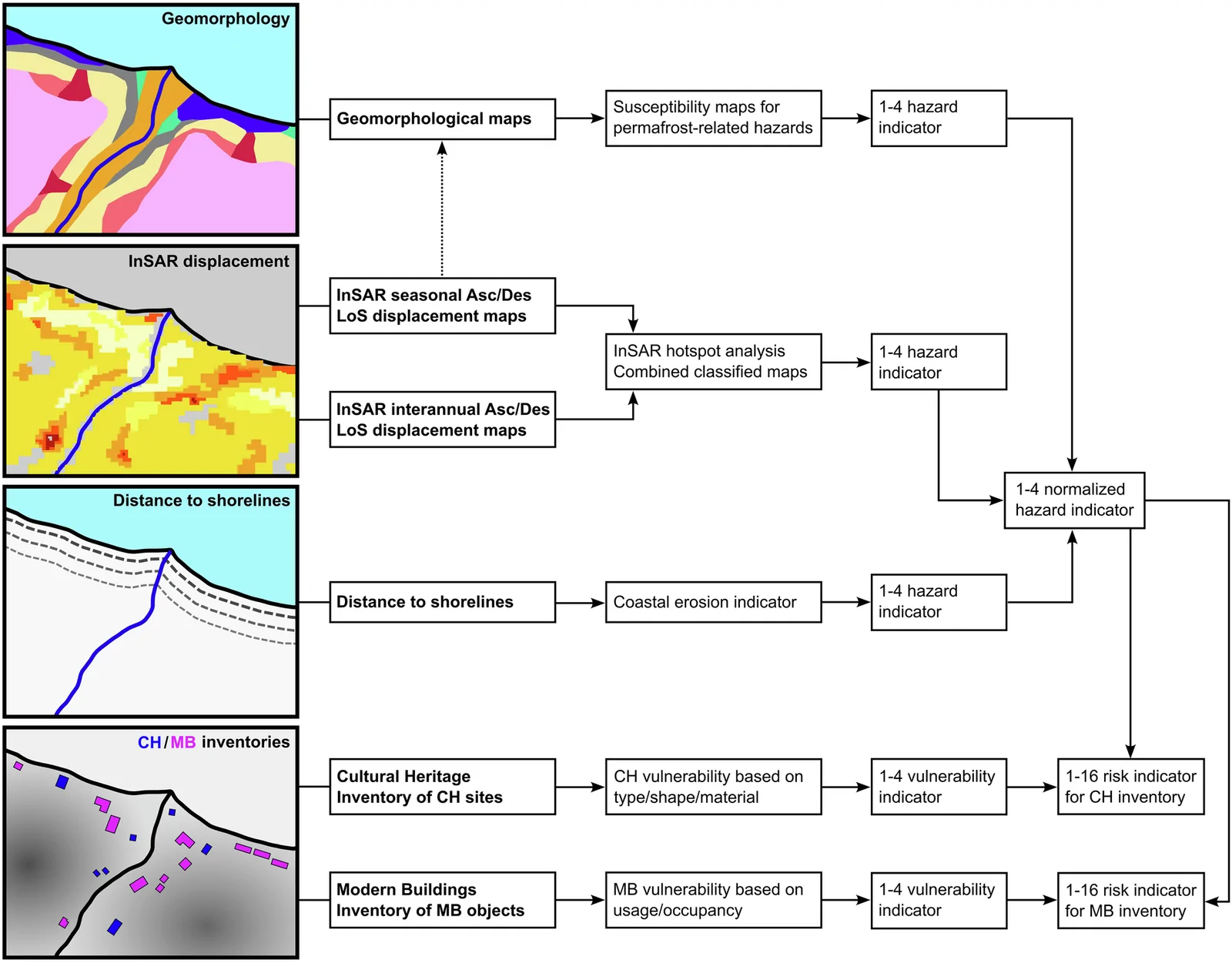
Methodological flowchart of the present study. Acronyms and abbreviations: Interferometric Synthetic Aperture Radar (InSAR), Ascending orbit (Asc), Descending orbit (Des), Line-of-Sight (LoS), Cultural Heritage (CH), Modern Buildings (MB).

Figure 2 highlights the methodological steps undertaken to obtain the qualitative hazard, vulnerability and risk scores for CH and MB.

### Combined hazard assessment

The hazard represents the physical phenomenon related to natural processes (e.g., coastal erosion, permafrost thaw) that has the potential to cause damage and loss to property, infrastructure, livelihoods, service provision and resources^69^. The combined hazard assessment was done by aggregating the information from three complementary hazard indicators. The scores were summed up and normalized (divided by three), to provide a similar categorisation in four qualitative hazard classes: 1 (low), 2 (low-medium), 3 (medium-high) to 4 (high).

### Geomorphological mapping

The geomorphological maps were constructed to fit a 1:30,000 scale, which was deemed to provide an adequate spatial and interpretational quality for the combination with InSAR movement data and the CH and MB objects under evaluation. The baseline data comprised orthorectified aerial photographs from year 2009 to 2011, supplied by the Norwegian Polar Institute (NPI)^70^. These aerial photographs were complemented by hillshade images and slope maps of the landscape, constructed from digital terrain models with minimum resolution of 5 m, also from the same source. The morphology and sediment cover was mapped and represents the last genetic process responsible for the deposit-formation. This classification was conducted in accordance with the Norwegian SOSI standard for polygons on Quaternary geological maps^71^. The only category that does not correspond to a genetically classified deposit is bare bedrock, which forms the baseline for all secondary sediment transformation (e.g., weathering), transportation (e.g., rock fall, glaciers, rivers) and depositional processes (e.g., lake sedimentation).

The maps were created using an integrated approach. Several pre-existing geomorphological or sedimentological maps from smaller or larger areas, derived from previous mapping or research projects^39–43,72–77^, were revised and remapped into a coherent legend-format and scale. Many of them had a higher level of detail than 1:30,000 scale, but had variable coverages and standard categories. In instances where geomorphological data was absent, the area was mapped directly. This was done by an operator (third author) having +15 years of field and mapping experience in central Svalbard. The results were peer-reviewed by an external colleague from the same field. The new maps have a detailed genetic subdivision of approximately 45 classes of depositional types and geomorphology stretching from: *i)* marine and fluvial/glaciofluvial process environments in the lower landscape, over the *ii)* glacial and pure permafrost morphologies (e.g., pingos and rock glaciers) and slope-dominated processes (e.g., rockfalls, snow avalanches and debris flows) to *iii) in situ* weathering and solifluction, periglacial processes which dominate low-angle areas on the mountain plateaus and lower slopes.

The final geomorphological maps were rearranged into eight geomorphological process classes according to their position in the landscape, the sedimentology (primarily grain-size composition related to the depositional environment), the permafrost-related interaction with surface and groundwater, the ground ice content and the expected processes leading to ground movement (Table 1). A hazard qualitative score was assigned to each geomorphological class, indicating its potential for destructive capacity on CH and MB, due to either ongoing surface processes (e.g., snow avalanches or landslides), sensitivity to frost heave, thaw subsidence and landform degradation (e.g., aeolian plains or degrading ice-cored moraines and pingos), or potential for direct damage or sediment burial from high-energy river processes.

**Table 1:** Geomorphological classes and the associated SOSI codes and hazard qualitative scores (hazard connected to permafrost-related ground movements) (*abbreviated names in italic refer to the field names in the dataset*).

Geomorphological Class (*GEOM_Class*)	Norwegian standard code (*SOSI_code)*	Hazard score (*H_Geom*)
1_Mass_Movement_Rapid	130, 301, 302, 307–311, 313–318	4
2_Mass_Movement_Slow	72, 320, 321	2
3_Complex_Internal_Deformation	88, 121	3
4_Ice_Loss_Degradation	1, 14, 15	4
5_Riverine_Processes	20, 50, 56	3
6_Heave_Subsidence_Plateaus	71	2
7_Heave_Subsidence_Lowland_Fine-grained	21, 22, 35, 51, 52, 41, 60, 61, 90, 207, 405	3
8_Heave_Subsidence_Lowland_Coarse-grained	11, 12, 42, 43, 46, 120, 122	1
9_Glacier	3	4

*SOSI codes are related to the specific sediment- and landform numeric codes in the official Norwegian SOSI standard for Quaternary Geological maps^71^.

### InSAR ground movement

The InSAR products builds on previous work to document ground dynamics in Svalbard^44,78^ using a Distributed Scatterer InSAR processing chain^79^. The second and fifth authors processed InSAR based on Sentinel-1 Interferometric Wide Swath modes snow-free images from 2016–2022 (ascending geometry, track 14 A) and 2018–2022 (descending geometry, track 154D). We used 8 to 19 images per season and geometry, i.e. in total 106 ascending images and 74 descending images in Longyearbyen and 96 ascending images and 71 descending images in Ny-Ålesund. The differences between the seasons and the areas are due to the variable data availability and duration of the thawing periods. We applied two different time intervals to build the image pairs (interferograms): (1) with 6 to 48 days during the thawing seasons, to represent the seasonal mean ground surface velocity (cm/summer); (2) with 340 to 390 days between the thawing seasons, to represent the interannual mean ground surface velocity (mm/year). Seasonal products typically document thaw subsidence from ground ice melt in the active layer and short-term mass movement on slopes. Interannual products typically document thaw subsidence from ground ice melt in the top of the permafrost and long-term mass movement on slopes. The distribution of low/high values in both products is correlated as it represents the ice-poor/ice-rich areas, which is related to the landform and sediment types^45^.

The initial results represent a multi-year averaged velocity with continuous values along the Line-of-Sight (LoS) of the radar sensor for each SAR geometry separately. The results are then combined and classified to simplify the information (hotspot identification)^80^. Ascending and descending results for both seasonal and interannual products are converted to absolute values and merged with a ‘maximum’ aggregate operation (GRASS *r.series* GIS module). The seasonal products are then classified into four classes of absolute velocity: 0–0.99, 1–1.99, 2–2.99, ≥3 cm/summer. The interannual products are classified into four classes of absolute velocity: 0–4, 4–8, 8–12, ≥12 mm/year. The velocity units (cm/summer and mm/year) are on purpose different for the two InSAR products to highlight the different magnitudes of the detected movement rates at the two different time scales (seasonal vs interannual). The chosen classes aim to provide an indication of low to high ground surface dynamics, associated with a hazard qualitative score (Table 2).

**Table 2:** Seasonal and interannual InSAR classes and associated qualitative hazard scores (*abbreviated names in italic refer to the field names in the dataset*).

InSAR seasonal velocity (cm/summer)	InSAR interannual velocity (mm/yr)	Ground surface dynamics classes	Hazard score (*H_InSAR)*
0–0.9999	0–3.9999	Low ground surface dynamics	1
1–1.9999	4–7.9999	Low-medium ground surface dynamics	2
2–2.9999	8–11.9999	Medium-high ground surface dynamics	3
≥3	≥12	High ground surface dynamics	4

The seasonal and interannual hazard scores are finally combined into a single ground surface dynamics hazard susceptibility map. When data was available for one product only (e.g., seasonal only), the value from the available product is documented. When data were available for both products (both seasonal and interannual), the scores were combined using an ‘average’ aggregate operation (GRASS *r.series* GIS module) and rounded up. For instance, if the pixel falls into class 3 for seasonal but class 1 for interannual, the combined result is 2. With a class 4 for seasonal but class 3 for interannual, the average is 3.5 and the combined score is rounded up to 4. The point is to summarise the InSAR information in one score that can be seen as the worst-case scenario. The final products are in a raster format (.tiff) with a 40 m resolution, due to SAR multi-looking (averaging to reduce the noise) and geocoding based on the NPI Digital Elevation Model (DEM)^81^.

### Coastal erosion

Coastal erosion estimates were based on previous studies from Svalbard^18,35,82,83^. High-resolution Planet SkySat satellite images were tasked for the two areas. For Longyearbyen, the image acquisition date is 17 July 2023, while for Ny-Ålesund, it is 03 September 2023^84^. The spatial resolution of the images is 0.5 × 0.5 m. One image was used for each area. The delineation of the coastline for both areas was made manually by the first and third authors. The coastline was then used to make buffer distances, as follows: 0–10 m, 10–20 m, 20–30 m, >30 m. Based on the most recent studies^18,35^, we consider the worst-case scenario of coastal erosion rates of −1 m/yr at a timescale of 30 years to set the upper-class limit (30 m). The hazard qualitative scores 4, 3, 2 and 1 were then attributed for each buffer distance (Table 3). In the absence of localised multi-year monitoring data along all coasts, we acknowledge this classification is a simplification. The objective is to emphasise the high climate sensitivity of the coastal areas in Svalbard.

**Table 3:** Coastal erosion buffers and associated qualitative hazard scores (*abbreviated names in italic refer to the field names in the dataset*).

Coastal erosion buffer distances (*distance*)	Coastal erosion hazard susceptibility classes	Hazard score (*H_Coastal*)
0–10 m	High coastal erosion hazard susceptibility	4
10–20 m	Medium-high coastal erosion hazard susceptibility	3
20–30 m	Low-medium coastal erosion hazard susceptibility	2
>30 m	Low coastal erosion hazard susceptibility	1

### Vulnerability assessment

The vulnerability represents the propensity or predisposition of a community, system, or asset to be adversely affected by a certain hazard^69^. The CH/MB vulnerability qualitative scores are based on Tables 4, 5. The results are kept separate for the two inventories (CH/MB) due to the different applied criteria and the variable expected uses of the results by different stakeholders.

**Table 4:** CH categories and associated vulnerability qualitative scores (*abbreviated names in italic refer to the field names in the dataset*).

CH category	Category	Examples of CH types	Vulnerability score (*V_CH*)
High standing structure	1	cableway support tower, crane, silo, lighthouse, mast	4
Low standing structure	2	building, house, garage, cabin, bridge, retaining wall	3
Foundation	3	turf foundation, trestle foundation, gun emplacement, mast base, cableway foundation	1
Track	4	route, road, railway, log bridge, pier/wharf, footbridge, cableway route	2
In ground installation	5	mine entrance/outlet/tunnel, pit	1
Loose find	6	fox trap, aircraft wreck part, cairn, wagon, hatch, pipe, pillar	3
Mine dump	7	rock/coal dump, storage area	1
Grave	8	grave	4

**Table 5:** MB categories and associated qualitative vulnerability scores (*abbreviated names in italic refer to the field names in the dataset*).

MB category	Examples of MB type	Vulnerability score (*V_MB*)
Buildings little occupied even during the day	Storage facility, garage	1
Buildings only occupied during the day, or low occupancy rate	Offices, restaurants, shops, or with low occupancy rate during the night (e.g., cabins)	2
Buildings occupied day and night, or with special value	Regular residence, hotels/lodges, or special cultural value not directly connected to human life (e.g., museum collection)	3
Buildings of high community relevance	Hospital, school/kindergarten, facilities used for emergency, evacuation, water and energy management	4

### CH types and classification

Based on the Norwegian Directorate for Cultural Heritage dataset locating and describing the CH sites^85^, as well as expert knowledge regarding CH assessment in Svalbard, eight categories were established based on each initial CH site description, according to its type, material and shape. After the categorisation, the next step was to assign each category a vulnerability qualitative score (Table 4), representing the expected sensitivity of the site to permafrost-related processes and ground movement.

There are differences in CH categories between Longyearbyen and Ny-Ålesund due to their distinct historical roles, developmental trajectories, and the types of human activities that have shaped each settlement over time (see more details in Table 1 of the Appendix in the Zenodo data package). Longyearbyen is a town with approx. 2500 inhabitants in 2026 and developed urban infrastructure. Cultural heritage has a large variety of types and categories, reflecting multiple periods of use and different historical backgrounds. In Ny-Ålesund, cultural heritage is subject to more stringent management and restricted general access. The CH sites are categorised around the themes “research” and “early mining”, corresponding to the two main activity eras of the settlement^51^.

### MB use and classification

The MB vulnerability assessment is based on the building type/usage and the interpretation in term of occupancy and exposure of local population. The buildings were categorised in four classes: (1) buildings little occupied, even during the day (e.g., storage facility, garage); (2) buildings only occupied during the day (e.g., offices, restaurants, shops), or with low occupancy rate during the night (e.g., cabins); (3) buildings occupied day and night (e.g., regular residence, hotels/lodges), or with special cultural/societal value not directly connected to human life (e.g., museum collection); (4) buildings of high community relevance (e.g., hospital, school/kindergarten, facilities used for emergency, evacuation, water and energy management) (Table 5). Some buildings remained undefined (score 0) when too little information on the usage was available to the authors.

The building footprints in Longyearbyen has been provided in a .shp format by the Longyearbyen Community Council (Longyearbyen Lokalstyre)^86^. The building footprints in Ny-Ålesund has been provided in a.shp format by NPI, KingsBay and the Norwegian Mapping Authority (Kartverket)^87^. In Longyearbyen, the initial dataset included an information about the building type, according to the Norwegian standard^88,89^. We automated the categorisation based on Table 5 for approx. 90% of the inventory. However, several polygons were undefined (approx. 7%), and some were outdated (approx. 2%). We therefore also accounted for our local knowledge for the vulnerability assessment. It consists in manual adjustments, both to reduce the number of undefined and to account for recent change of usage (e.g., new hotels). In Ny-Ålesund, the database did not include any information on building type and usage. We therefore based our vulnerability assessment on our local knowledge and complementary online resources^90,91^. Some polygons are overlapping between CH and MB inventories for both Longyearbyen and Ny-Ålesund, because several CH buildings are still used in the present day. We keep both information because the vulnerability is assessed differently (type/material/historical value vs usage/occupancy/exposure of population) and expected to be used differently by various stakeholders with variable objectives (CH protection, MB management, areal planning, etc.).

### Risk assessment

**Fig. 3:**
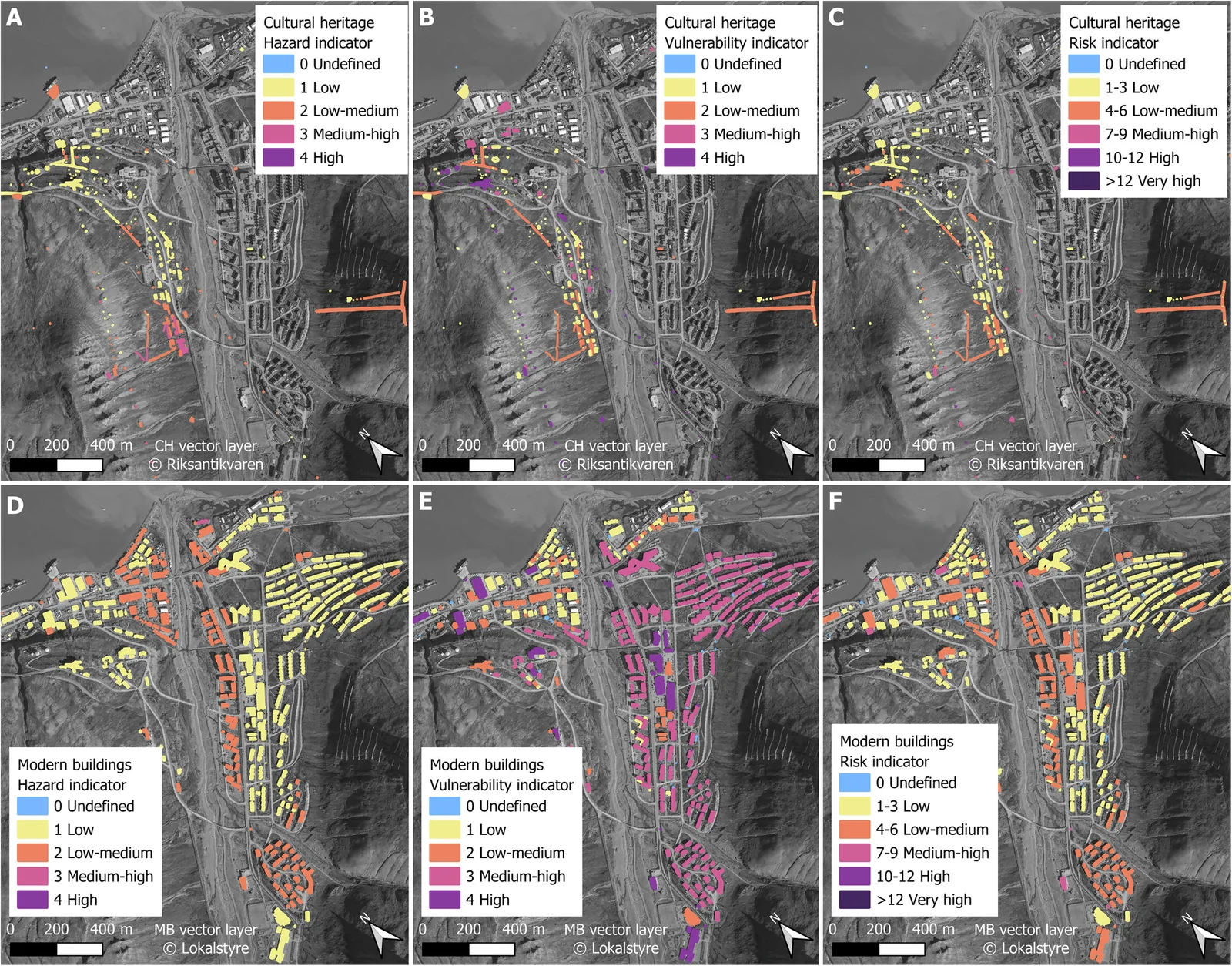
Example of mapped results of the hazard, vulnerability and risks indicators in Longyearbyen. Note that the maps are only for a selected zoom in a subarea and therefore do not show all results. (**A–C**) Cultural heritage inventory. (D–F) Modern building inventory. A & D. Hazard indicator. B & E. Vulnerability indicator. C & F. Risk indicator.

**Fig. 4:**
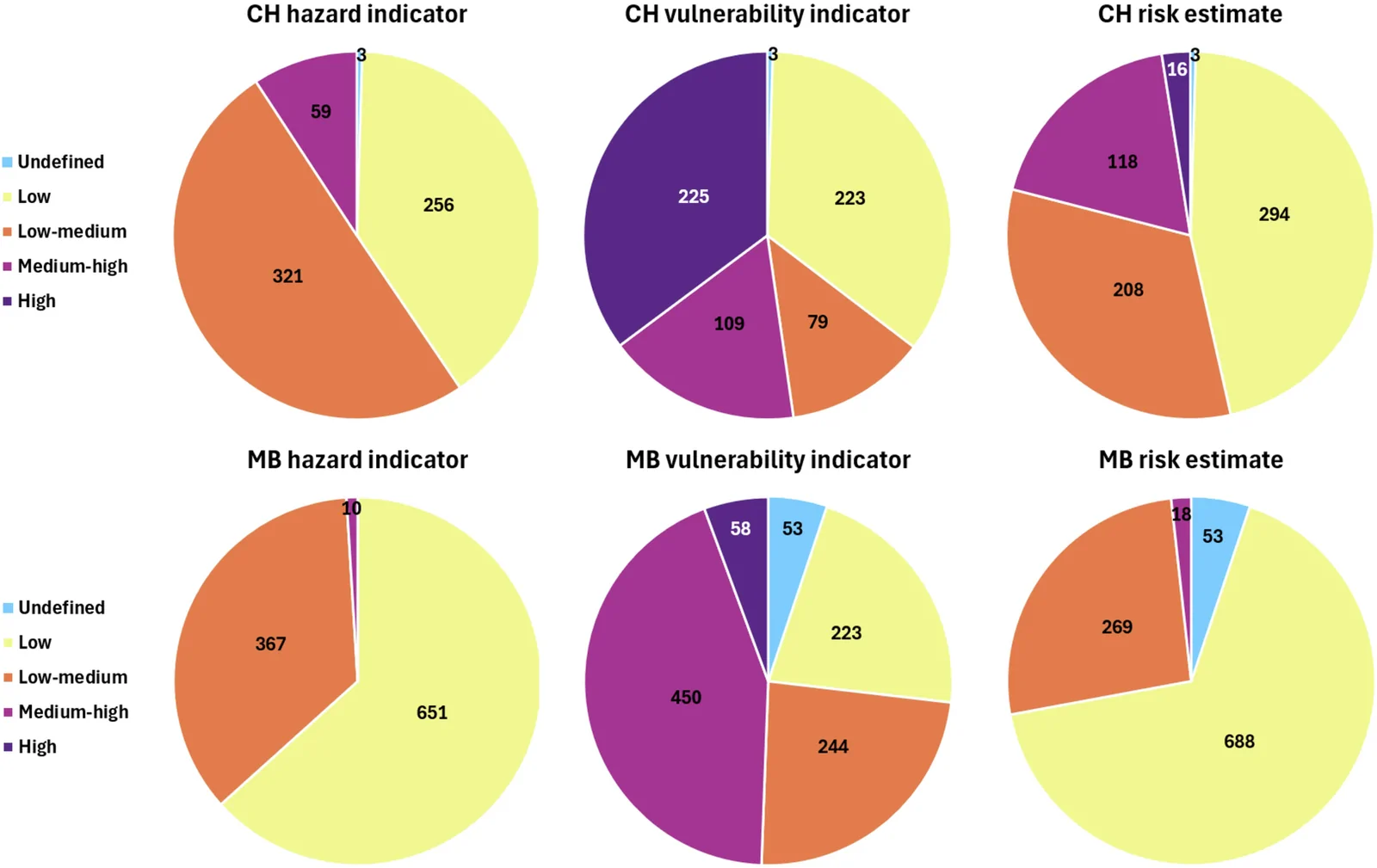
Distribution of hazard and vulnerability indicators and combined risk estimate for Cultural Heritage (CH) and Modern Buildings (MB) in Longyearbyen (the whole area). This example follows the risk categorisation suggested in Fig. 3 (0 = undefined, 1–3 = low, 4–6 = low-medium, 7–9 = medium-high, 10–12 = high, >12 = very high).

**Fig. 5:**
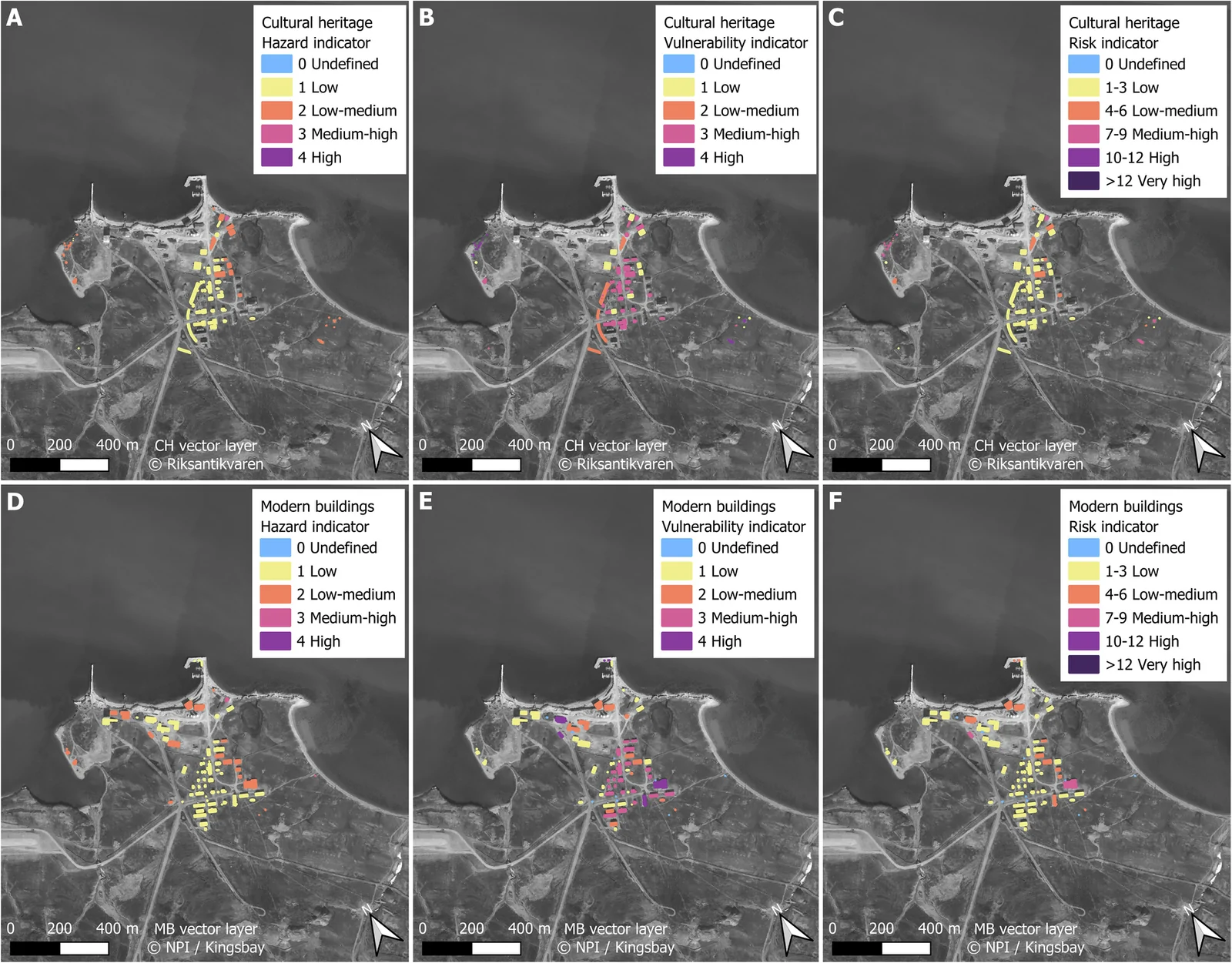
Example of mapped results of the hazard, vulnerability and risks indicators in Ny-Ålesund. Note that the maps are only for a selected zoom in a subarea and therefore do not show all results. (**A–C**) Cultural Heritage inventory. (**D–F**) Modern Building inventory. A & D. Hazard indicator. B & E. Vulnerability indicator. C & F. Risk indicator.

**Fig. 6:**
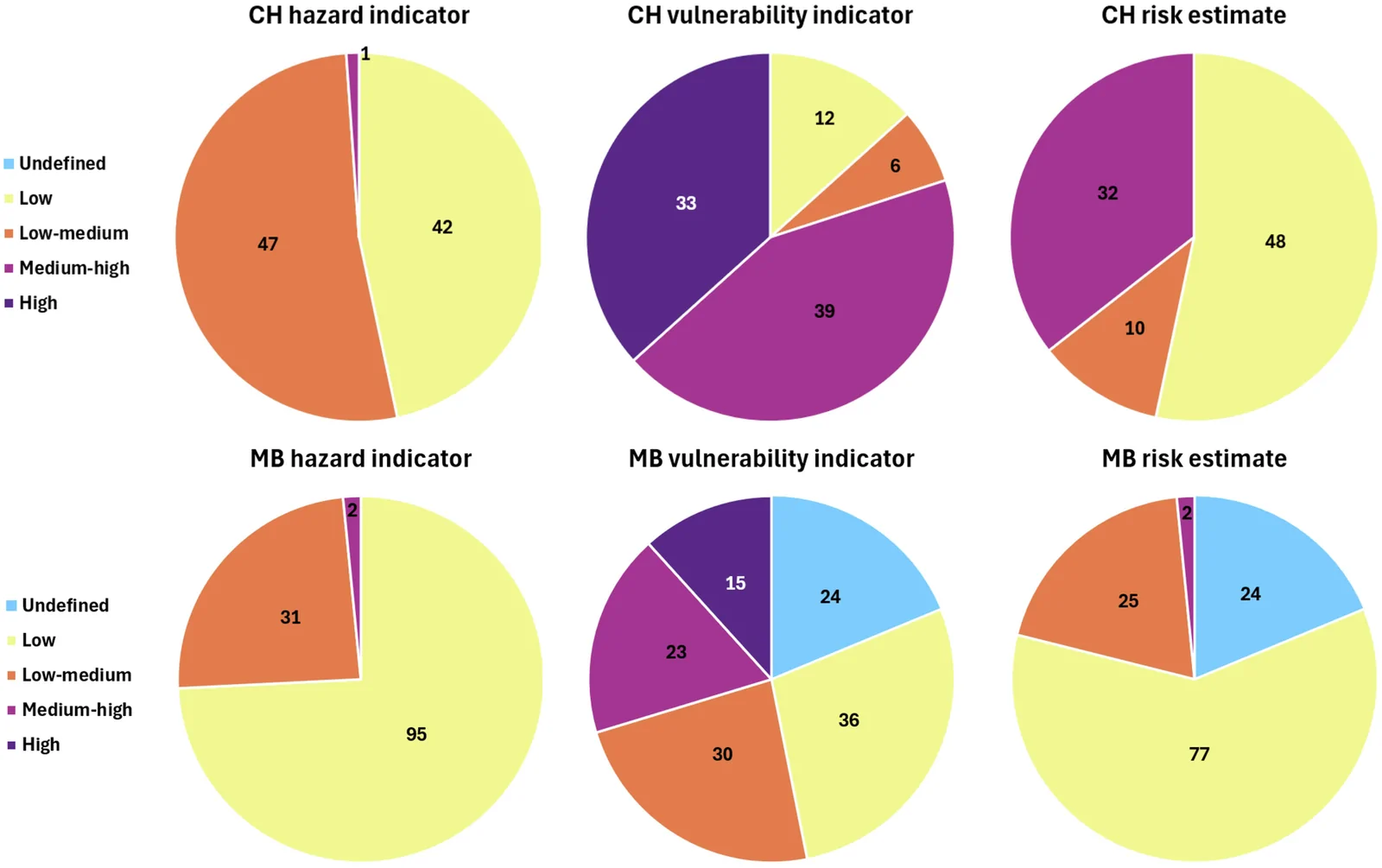
Distribution of hazard and vulnerability indicators and combined risk estimate for Cultural Heritage (CH) and Modern Buildings (MB) in Ny-Ålesund (the whole area). This example follows the risk categorisation suggested in Fig. 5 (0 = undefined, 1–3 = low, 4–6 = low-medium, 7–9 = medium-high, 10–12 = high, >12 = very high).

The risk represents potential consequences of hazard events on the investigated areas and exposed elements (people or objects). It can be expressed in probabilistic, (semi-)quantitative or qualitative terms^69^. In our study, the risk is documented qualitatively and provides relative information about the level of potential impacts to the inventoried CH/MB. The risk is the product of the hazard and vulnerability indicators. The multiplicative form is an established convention to avoid providing a result other than zero in case one of the components of the risk calculation remains undefined^92,93^. The product also reflects that the risk is the complex and nonlinear interaction of the natural and human components, not a simple summation. The two components interact conditionally, which implies a multiplicative behaviour. The combined hazard score ranges from 0 to 4 (0 for undefined) and the vulnerability is between 0 to 4 (0 for undefined). Therefore, the risk estimate ranges from 0 to 16 (0 for undefined). The users can then choose further ways for categorisation and visual representation on maps or with graphs, depending on their objectives. Figures 3–6 show examples of how the results could be used for identifying locations with the highest (relative) risk, to be used for prioritisation of adaptation or mitigation measures.

## Data Records

The dataset is available for download in the Zenodo repository^94^ (https://doi.org/10.5281/zenodo.18154592), and consists of a series of geospatial files that can be imported into any GIS environment. The dataset includes vector (polygons) and raster files in shapefile (.shp) and GeoTIFF (.tiff) formats.

There are two main folders, corresponding to the two study areas: “Longyearbyen_LYB” and “Ny-Aalesund_NYA”. Each of the two main folders has five subfolders, compiling the diverse intermediate and final products: “1_Geomorphological_map_LYB/NYA”, “2_InSAR_ground_movement_LYB/NYA”, “3_Coastal_erosion_LYB/NYA”, “4_Cultural_heritage_LYB/NYA” and “5_Modern_buildings_LYB/NYA”. Each folder contains the vector/raster files and an Appendix (metadata, detailed information about each attribute/field of the data contained in the respective folder).

## Technical Validation

The database contains 639 CH sites and 1028 MB in Longyearbyen, and 90 CH sites and 128 MB in Ny-Ålesund. The distribution of the hazard indicators for CH and MB is summarised in Tables 6–9. Examples of visualisation of the normalized hazard, vulnerability and risk indicators as maps or pie charts are shown in Figs. 3–6.

**Table 6:** No. of CH sites falling in the different categories of geomorphological (*H_Geom*), InSAR (*H_InSAR*) and coastal erosion (*H_Coastal*) hazard scores and resulting normalized hazard scores (*H_norm*) in Longyearbyen.

H_Geom	No. of CH (Geom)	H_InSAR	No. of CH (InSAR)	H_Coastal	No. of CH (Coastal)	H_norm
4	128	4	26	4	10	4
3	114	3	88	3	4	3
2	204	2	260	2	1	2
1	190	1	262	1	621	1
NULL	3	NULL	3	NULL	3	NULL
**Total**	**639**		**639**		**639**

**Table 7:** No. of CH sites falling in the different categories of geomorphological (*H_Geom*), InSAR (*H_InSAR*) and coastal erosion (*H_Coastal*) hazard scores and resulting normalized hazard scores (*H_norm*) in Ny-Ålesund.

H_Geom	No. of CH (Geom)	H_InSAR	No. of CH (InSAR)	H_Coastal	No. of CH (Coastal)	H_norm
4	0	4	0	4	10	4
3	3	3	0	3	11	3
2	83	2	22	2	4	2
1	4	1	68	1	65	1
Total	**90**		**90**		**90**

**Table 8:** No. of MB falling in the different categories of geomorphological (*H_Geom*), InSAR (*H_InSAR*) and coastal erosion (*H_Coastal*) hazard scores and resulting normalized hazard scores (*H_norm*) in Longyearbyen.

H_Geom	No. of MB (Geom)	H_InSAR	No. of MB (InSAR)	H_Coastal	No. of MB (Coastal)	H_norm
4	75	4	3	4	44	4
3	181	3	17	3	23	3
2	164	2	400	2	21	2
1	608	1	608	1	940	1
Total	**1028**		**1028**		**1028**

**Table 9:** No. of MB falling in the different categories of geomorphological (*H_Geom*), InSAR (*H_InSAR*) and coastal erosion (*H_Coastal*) hazard scores and resulting normalized hazard scores (*H_norm*) in Ny-Ålesund.

H_Geom	No. of MB (Geom)	H_InSAR	No. of MB (InSAR)	H_Coastal	No. of MB (Coastal)	H_norm
4	0	4	0	4	7	4
3	2	3	1	3	5	3
2	88	2	27	2	0	2
1	38	1	100	1	116	1
Total	**128**		**128**		**128**	

### Dataset limitations

Several technical limitations must be mentioned due to the approach and diverse methods applied to compile the dataset. The geomorphological mapping was based on the orthophotos from NPI^70^ from 2009–2011; these were complemented by substantial knowledge of the local landscape and landforms of the third and sixth authors. Coastal erosion buffer distances were based on the Planet data from July and September 2023^84^. Data users must consider that the landscape might have changed since the source dates and will keep evolving in the future.

The InSAR processing was performed with the objective to develop simple hotspot maps, highlighting the areas with ground surface dynamics, in a relative manner. InSAR is a measurement technique suited for gradual and relatively slow movement. When the changes are too sudden or the surface is too wet, the InSAR signal stability is lost, and no information is available. It is for instance not suited for mapping quick shallow landslides or rockfall. The InSAR maps must therefore be seen as a tool complementary to geomorphological mapping. The InSAR results have a 40 m resolution, i.e., they are suitable to identify quite localised movement patterns, but the movement source can be mixed if several processes occur within a 40 m pixel. The movement rate corresponds to the average of several years (2016–2022), so the results are not suited to detect very recent movement processes.

For CH sites, positional accuracy might vary slightly, as objects were digitised from different source materials and at different times, including small-scale map sources, generalised geometries, or legacy registrations. Another limitation relates to the attribute information, such as dating, function, value, or preservation status: some fields were missing or inconsistently populated. Vulnerability scores are qualitative and may change over time, reflecting assessments of potential for damage, depending on the material/type. The national cultural heritage database^85^ is dynamic and updated over time, representing a temporal snapshot; any subsequent changes in site status, geometry, or classification are not captured and may cause discrepancies for users working with newer versions. This dataset was downloaded on 1 February 2024.

The same temporal consideration applies to the MB inventory, provided by the Longyearbyen Community Council (Longyearbyen Lokalstyre) on 21 February 2025 for Longyearbyen, and by NPI on 19 August 2025 for Ny-Ålesund (personal communications)^86,87^. Any potential later updates of the initial datasets are not included in our current results. In Longyearbyen, several newly built objects were missing in the inventory that was used. The vulnerability assessment required manual categorisation based on local knowledge of the authors, due to missing information on the building type in the databases. In both areas, we acknowledge the subjectivity of the assessment and emphasize that the categorisation is indicative and might be adjusted by end-users with complementary or updated knowledge in the future.

Overall, the hazard, vulnerability and risk scores provided in the dataset are qualitative examples, based on expert input and scientific literature, and are intended to demonstrate possible methodologies rather than prescribe definitive values. Users are encouraged to adapt the scoring schemes to suit their own context, data availability, and management priorities. The scoring and prioritisation methods presented serve as decision-support tools for local authorities responsible for CH management and MB planning. However, for other Arctic and cold regions, it may be necessary to recalibrate indices, criteria, or data layers to meet specific regional requirements. All scoring and prioritisation examples are intended to support, rather than dictate, risk-informed decision-making in CH and MB preservation and maintenance.

## Data Availability

The dataset is available for download in the Zenodo repository^94^ (https://doi.org/10.5281/zenodo.18154592). It is open and available to all researchers, users and authorities. The entire process has been carried out in ArcGIS v10.6.1, but all items have been produced in a format that can be imported into any GIS environment.

## Code availability

No customised code was produced to create or analyse this dataset. We used ArcGIS v10.6.1 software to create and process the data. The dataset can be opened in any GIS software (open-sourced or licensed).

## References

[1] Biskaborn, B. K. *et al*. Permafrost is warming at a global scale. *Nature Communications* **10**, 264, 10.1038/s41467-018-08240-4 (2019). PMC6335433 30651568

[2] Bjerke, J. W. *et al*. Nordic boreo-arctic lands under rapid climatic change: A review of recent and future trends and extreme events. *Earth-Science Reviews* **261**, 105012, 10.1016/j.earscirev.2024.105012 (2024).

[3] Smith, S. L., O’Neill, H. B., Isaksen, K., Noetzli, J. & Romanovsky, V. E. The changing thermal state of permafrost. *Nature Reviews Earth & Environment* **3**, 10–23, 10.1038/s43017-021-00240-1 (2022).

[4] Strand, S. M. & Christiansen, H. H. Permafrost thermal regime and active layer dynamics across periglacial landforms in Svalbard’s Nordenskiöld Land Permafrost Observatory, 2008–2023. *Arctic Science* **11**, 1–19, 10.1139/as-2024-0027 (2025).

[5] Chen, J., Liu, L., Zhang, T., Cao, B. & Lin, H. Using Persistent Scatterer Interferometry to Map and Quantify Permafrost Thaw Subsidence: A Case Study of Eboling Mountain on the Qinghai-Tibet Plateau. *Journal of Geophysical Research: Earth Surface* **123**, 2663–2676, 10.1029/2018jf004618 (2018).

[6] O’Neill, H. B., Smith, S. L., Burn, C. R., Duchesne, C. & Zhang, Y. Widespread Permafrost Degradation and Thaw Subsidence in Northwest Canada. *Journal of Geophysical Research: Earth Surface* **128**, e2023JF007262, 10.1029/2023jf007262 (2023).

[7] Hjort, J. *et al*. Impacts of permafrost degradation on infrastructure. *Nature Reviews Earth & Environment* **3**, 24–38, 10.1038/s43017-021-00247-8 (2022).

[8] Eichel, J. *et al*. Unmanned aerial vehicle‐based mapping of turf‐banked solifluction lobe movement and its relation to material, geomorphometric, thermal and vegetation properties. *Permafrost and Periglacial Processes* **31**, 97–109, 10.1002/ppp.2036 (2020).

[9] Harkema, M. R., Nijland, W., de Jong, S. M., Kattenborn, T. & Eichel, J. Monitoring solifluction movement in space and time: A semi-automated high-resolution approach. *Geomorphology* **433**, 108727, 10.1016/j.geomorph.2023.108727 (2023).

[10] Rubensdotter, L., Nicu, I. C. & Stalsberg, K. Complex geohazards at a high priority Arctic cultural heritage site at Russekeila – Kapp Linné, Svalbard. *Catena* **254**, 108935, 10.1016/j.catena.2025.108935 (2025).

[11] Nicu, I. C., Rubensdotter, L., Tanyas, H. & Lombardo, L. First retrogressive thaw slump (RTS) inventory for the Kanin Peninsula (NW Russia). *Scientific Data* **12**, 1221, 10.1038/s41597-025-05592-7 (2025). PMC12259900 40659663

[12] Nitze, I. *et al*. A Labeling Intercomparison of Retrogressive Thaw Slumps by a Diverse Group of Domain Experts. *Permafrost and Periglacial Processes* **36**, 83–92, 10.1002/ppp.2249 (2024).

[13] Thomas, M. *et al*. Evidence for preservation of organic carbon interacting with iron in material displaced from retrogressive thaw slumps: Case study in Peel Plateau, western Canadian Arctic. *Geoderma* **433**, 116443, 10.1016/j.geoderma.2023.116443 (2023).

[14] Yang, Y. *et al*. A Collaborative and Scalable Geospatial Data Set for Arctic Retrogressive Thaw Slumps with Data Standards. *Scientific Data* **12**, 18, 10.1038/s41597-025-04372-7 (2025). PMC11704310 39762331

[15] Nicu, I. C., Tanyas, H., Rubensdotter, L. & Lombardo, L. A glimpse into the northernmost thermo-erosion gullies in Svalbard archipelago and their implications for Arctic cultural heritage. *Catena* **212**, 106105, 10.1016/j.catena.2022.106105 (2022).

[16] Perreault, N., Lévesque, E., Fortier, D., Gratton, D. & Lamarque, L. J. Remote sensing evaluation of High Arctic wetland depletion following permafrost disturbance by thermo-erosion gullying processes. *Arctic Science* **3**, 237–253, 10.1139/as-2016-0047 (2017).

[17] Perreault, N., Lévesque, E., Fortier, D. & Lamarque, L. J. Thermo-erosion gullies boost the transition from wet to mesic tundra vegetation. *Biogeosciences* **13**, 1237–1253, 10.5194/bg-13-1237-2016 (2016).

[18] Nicu, I. C., Rubensdotter, L., Stalsberg, K. & Nau, E. Coastal Erosion of Arctic Cultural Heritage in Danger: A Case Study from Svalbard, Norway. *Water* **13**, 784, 10.3390/w13060784 (2021).

[19] Nielsen, D. M. *et al*. Increase in Arctic coastal erosion and its sensitivity to warming in the twenty-first century. *Nature Climate Change* **12**, 263–270, 10.1038/s41558-022-01281-0 (2022).

[20] Tanguy, R. *et al*. Pan‐Arctic Assessment of Coastal Settlements and Infrastructure Vulnerable to Coastal Erosion, Sea‐Level Rise, and Permafrost Thaw. *Earth’s Future* **12**, e2024EF005013, 10.1029/2024ef005013 (2024).

[21] Suzuki, K. *et al*. Hydrological Variability and Changes in the Arctic Circumpolar Tundra and the Three Largest Pan-Arctic River Basins from 2002 to 2016. *Remote Sensing* **10**, 402, 10.3390/rs10030402 (2018).

[22] Patro, E. R. *et al*. Flow regime alteration in Arctic rivers due to dam operations and climate change. *Global and Planetary Change* **237**, 104442, 10.1016/j.gloplacha.2024.104442 (2024).

[23] Bring, A., Shiklomanov, A. & Lammers, R. B. Pan-Arctic river discharge: Prioritizing monitoring of future climate change hot spots. *Earth’s Future* **5**, 72–92, 10.1002/2016ef000434 (2017).

[24] Manos, E., Witharana, C. & Liljedahl, A. K. Permafrost thaw-related infrastructure damage costs in Alaska are projected to double under medium and high emission scenarios. *Communications Earth & Environment* **6**, 221, 10.1038/s43247-025-02191-7 (2025).

[25] Jin, X.-Y. *et al*. Impacts of climate-induced permafrost degradation on vegetation: A review. *Advances in Climate Change Research* **12**, 29–47, 10.1016/j.accre.2020.07.002 (2021).

[26] Peng, X. *et al*. Active Layer Thickness and Permafrost Area Projections for the 21st Century. *Earth’s Future* **11**, e2023EF003573, 10.1029/2023ef003573 (2023).

[27] Rantanen, M. *et al*. The Arctic has warmed nearly four times faster than the globe since 1979. *Communications Earth & Environment* **3**, 168, 10.1038/s43247-022-00498-3 (2022).

[28] Førland, E. J. *et al*. Measured and Modeled Historical Precipitation Trends for Svalbard. *Journal of Hydrometeorology* **21**, 1279–1296, 10.1175/jhm-d-19-0252.1 (2020).

[29] Bartsch, A. *et al*. Expanding infrastructure and growing anthropogenic impacts along Arctic coasts. *Environmental Research Letters* **16**, 115013, 10.1088/1748-9326/ac3176 (2021).

[30] Nicu, I. C. & Fatorić, S. Climate change impacts on immovable cultural heritage in polar regions: A systematic bibliometric review. *WIREs Climate Change* **14**, e822, 10.1002/wcc.822 (2023).

[31] Nicu, I. C., Elia, L., Rubensdotter, L., Tanyas, H. & Lombardo, L. Multi-hazard susceptibility mapping of cryospheric hazards in a high-Arctic environment: Svalbard Archipelago. *Earth System Science Data* **15**, 447–464, 10.5194/essd-15-447-2023 (2023).

[32] Nicu, I. C., Guzman, P. & Stoleriu, C. C. Unfreezing the past: near Pan-Svalbard assessment of cryospheric hazards to Arctic cultural heritage. *Science of the Total Environment* **1000**, 180424, 10.1016/j.scitotenv.2025.180424 (2025). 40914113

[33] Wieczorek, I., Nicu, I. C., Swirad, Z. M. & Strzelecki, M. C. Documenting an isolated and high-priority Arctic cultural heritage site: case study from Kapp Pettersen, Svenskøya, Svalbard. *Polar Research* **45**, 12605, 10.33265/polar.v45.12605 (2026).

[34] Nicu, I. C., Lombardo, L. & Rubensdotter, L. Preliminary assessment of thaw slump hazard to Arctic cultural heritage in Nordenskiöld Land, Svalbard. *Landslides* **18**, 2935–2947, 10.1007/s10346-021-01684-8 (2021).

[35] Nicu, I. C., Stalsberg, K., Rubensdotter, L., Martens, V. V. & Flyen, A.-C. Coastal Erosion Affecting Cultural Heritage in Svalbard. A Case Study in Hiorthhamn (Adventfjorden)—An Abandoned Mining Settlement. *Sustainability* **12**, 2306, 10.3390/su12062306 (2020).

[36] Hjort, J. *et al*. Degrading permafrost puts Arctic infrastructure at risk by mid-century. *Nature Communications* **9**, 5147, 10.1038/s41467-018-07557-4 (2018). PMC6289964 30538247

[37] Karjalainen, O. *et al*. Circumpolar permafrost maps and geohazard indices for near-future infrastructure risk assessments. *Scientific Data* **6**, 190037, 10.1038/sdata.2019.37 (2019). PMC6413688 30860499

[38] Scheer, J., Tomaškovičová, S. & Ingeman-Nielsen, T. Thaw settlement susceptibility mapping for roads on permafrost - Towards climate-resilient and cost-efficient infrastructure in the Arctic. *Cold Regions Science and Technology* **220**, 104136, 10.1016/j.coldregions.2024.104136 (2024).

[39] Tolgensbakk, J., Sørbel, L. & Høgvard, K. *Geomorphological and Quaternary Geological Map of Svalbard 1:100,000. Sheet C9Q Adventdalen* [map]. Norwegian Polar Institute (NPI), www.nb.no/items/89c04162040ba882108df476db5082bc?page=0 (2001).

[40] Miccadei, E., Piacentini, T. & Berti, C. Geomorphological features of the Kongsfjorden area: Ny-Ålesund, Blomstrandøya (NW Svalbard, Norway). *Rendiconti Lincei* **27**, 217–228, 10.1007/s12210-016-0537-3 (2016).

[41] Berthling, I. *et al*. Analysis of the paraglacial landscape in the Ny-Ålesund area and Blomstrandøya (Kongsfjorden, Svalbard, Norway). *Journal of Maps* **16**, 818–833, 10.1080/17445647.2020.1837684 (2020).

[42] Rubensdotter, L., Larsen, E. & Lyså, A. *Kvartærgeologisk og geomorfologisk kart, Svea, Svalbard / Quaternary geological and geomorphological map, Svea, Svalbard. M 1:15 000* [map]. Geological Survey of Norway (NGU), www.ngu.no/publikasjon/kvartaergeologisk-og-geomorfologisk-kart-svea-svalbard-quaternary-geological-and (2016)

[43] Rubensdotter, L., Stalsberg, K., Christiansen, H. H., Eckerstorfer, M. & Trøyen, P. *Landforms and sediments in Todalen and upper Gangdalen and Bødalen, Svalbard. Scale 1:25 000* [map]. Geological Survey of Norway (NGU), www.ngu.no/en/node/8616 (2015).

[44] Rouyet, L., Lauknes, T. R., Christiansen, H. H., Strand, S. M. & Larsen, Y. Seasonal dynamics of a permafrost landscape, Adventdalen, Svalbard, investigated by InSAR. *Remote Sensing of Environment* **231**, 111236, 10.1016/j.rse.2019.111236 (2019).

[45] Wendt, L., Rouyet, L., Christiansen, H. H., Lauknes, T. R. & Westermann, S. InSAR sensitivity to active layer ground ice content in Adventdalen, Svalbard. *The Cryosphere* **20**, 1179–1197, 10.5194/tc-20-1179-2026 (2026).

[46] Makopoulou, E. *et al*. Retrogressive thaw slump susceptibility in the northern hemisphere permafrost region. *Earth Surface Processes and Landforms* **49**, 3319–3331, 10.1002/esp.5890 (2024).

[47] Makopoulou, E., Karjalainen, O., Lipovsky, P., Blais-Stevens, A. & Hjort, J. Susceptibility of active-layer detachment failures and vulnerability of infrastructure in Alaska and northwestern Canada. *Landslides* **22**, 3561–3575, 10.1007/s10346-025-02603-x (2025).

[48] Chen, W. *et al*. Mapping the susceptibility to freeze-thaw deterioration and regionalization of freeze-thaw environments of earthen sites in China: A preliminary study. *Science of the Total Environment* **995**, 176995, 10.1016/j.scitotenv.2024.176995 (2024). 39427889

[49] Governor of Svalbard (Sysselmesteren på Svalbard).*Kulturminneplan for Svalbard 2013 – 2023*[Plan for the management of cultural heritage in Svalbard 2013 – 2023]. Report Series Nr. 1/2013, 112 pp., https://www.sysselmesteren.no/contentassets/bffbcd7fa7ae42ad8c6c22f047b360b3/kulturminneplan-2013–2023.pdf (2013)

[50] Bekele, Y. & Sinitsyn, A. O. Impact of Climate Change on Infrastructure in Longyearbyen. Case Study of Pile Foundations on Sloping Terrains. *SINTEF Notes* **36**, 51 pp., 10.13140/RG.2.2.33278.33601 (2020).

[51] Aga, J. *et al*. Impact of snow and building management on ground surface temperatures in permafrost environments - A case study from the historical mining town Ny-Ålesund, Svalbard. *Cold Regions Science and Technology* **237**, 104516, 10.1016/j.coldregions.2025.104516 (2025).

[52] Ravan, M., Revez, M. J., Pinto, I. V., Brum, P. & Birkmann, J. A Vulnerability Assessment Framework for Cultural Heritage Sites: The Case of the Roman Ruins of Tróia. *International Journal of Disaster Risk Science* **14**, 26–40, 10.1007/s13753-023-00463-4 (2023).

[53] Vaguet, Y., Ourng, C. & Moriconi-Ebrard, F. Is the Arctic really a frontier? Settlement dynamics within various Arctic Limits based on the Arcticapolis geohistorical database. *Espace Populations Sociétés*, 10.4000/eps.10277 (2021).

[54] Bonusiak, G. Development of Ecotourism in Svalbard as Part of Norway’s Arctic Policy. *Sustainability* **13**, 962, 10.3390/su13020962 (2021).

[55] Biresselioglu, M. E., Demir, M. H., Solak, B., Kayacan, A. & Altinci, S. Investigating the trends in arctic research: The increasing role of social sciences and humanities. *Science of the Total Environment* **729**, 139027, 10.1016/j.scitotenv.2020.139027 (2020). 32498176

[56] Werner, K. *et al*. Arctic in Rapid Transition: Priorities for the future of marine and coastal research in the Arctic. *Polar Science* **10**, 364–373, 10.1016/j.polar.2016.04.005 (2016).

[57] Guzman, P. Cultural heritage in climate planning: An analysis of the Norwegian national climate documents and guidelines. *Journal of Cultural Heritage* **74**, 35–47, 10.1016/j.culher.2025.05.003 (2025).

[58] LPO Svalbard. *Kulturhistorisk stedsanalyse for Longyearbyen* [Cultural historical site analysis for Longyearbyen]*.* LPO Arkitekter AS, 112 pp., https://www.lpo.no/assets/Rapport_Kulturhistorisk-stedsanalyse-for-Longyearbyen.pdf (2025).

[59] Bekele, Y. & Sinitsyn, A. O. *Risk Analysis of the Impact of Natural Hazards on Cultural Heritage. Development of a Risk Assessment Tool.* PCCH-Arctic Report Nr. 2. SINTEF Report 2023:00250, ISBN 978-82-14-07953-1, 57 pp., https://www.sintef.no/contentassets/cee45ce80f54436f98eb86fcc9e52b24/pcch-arctic-report-r2---updated-with-doi---signed.pdf (2023).

[60] Guzman, P. & Daly, C. Integrating cultural resources and heritage in climate action: A review of nine climate plans. *Environmental Science & Policy* **171**, 104127, 10.1016/j.envsci.2025.104127 (2025).

[61] Ran, Y. *et al*. Permafrost degradation increases risk and large future costs of infrastructure on the Third Pole. *Communications Earth & Environment* **3**, 238, 10.1038/s43247-022-00568-6 (2022).

[62] Aktürk, G. A systematic overview of the barriers to building climate adaptation of cultural and natural heritage sites in polar regions. *Environmental Science & Policy* **136**, 19–32, 10.1016/j.envsci.2022.05.016 (2022).

[63] Biesbroek, R., Mirbach, C., Sietsma, A. & Garschagen, M. Assessment of existing datasets for tracking progress towards the Global Goal on Adaptation (and beyond). *Climate Policy* **26**, 535–549, 10.1080/14693062.2025.2517644 (2025).

[64] Meyer, A. Physical and feasible: Climate change adaptation in Longyearbyen, Svalbard. *Polar Record* **58**, e29, 10.1017/s0032247422000079 (2022).

[65] Nicu, I. C., Granberg, M. & Undall, E. Short overview on international historic climate adaptation of built heritage to natural hazards: lessons for Norway. *International Journal of Conservation Science* **13**, 441–456, https://ijcs.ro/public/IJCS-22-32_Nicu.pdf (2022).

[66] Varnajot, A. & Makopoulou, E. Tourism in the Arctic is at risk due to intensifying permafrost degradation. *Communications Earth & Environment* **6**, 983, 10.1038/s43247-025-02944-4 (2025).

[67] Zwoliński, Z. *et al*. Geomorphological settings of Polish research areas on Spitsbergen. *Landform Analysis* **22**, 125–143, 10.12657/landfana.022.011 (2013).

[68] Johannessen, S., Hancock, H., Wickström, S. & Albrechtsen, E. Risk governance of climate-related hazards in Longyearbyen, Svalbard: A review of risk governance approaches and knowledge gaps. *Climate Risk Management* **43**, 100585, 10.1016/j.crm.2024.100585 (2024).

[69] Gallina, V. *et al*. A review of multi-risk methodologies for natural hazards: Consequences and challenges for a climate change impact assessment. *Journal of Environmental Management* **168**, 123–132, 10.1016/j.jenvman.2015.11.011 (2016). 26704454

[70] Norwegian Polar Institute (Norsk Polarinstitutt, NPI). *Svalbard Orthophoto*, https://geodata.npolar.no/arcgis/rest/services/Basisdata/NP_Ortofoto_Svalbard_WMTS_25833/MapServer/WMTS/1.0.0/WMTSCapabilities.xml (2026).

[71] Geological Survey of Norway (Norges Geologiske Undersøkelse, NGU). *Produktspesifikasjon: ND_Løsmasser* [Product specification: Superficial deposits], https://www.ngu.no/upload/Aktuelt/Produktspesifikasjon_LosmasseN250_N50.pdf (2010).

[72] Sassenroth, C. V., Salvatore, M. C., Hauber, E., Bucher, T. & Baroni, C. Geomorphological map of Kvadehuksletta, Brøggerhalvøya (Svalbard, Norway). *Geografia Fisica e Dinamica Quaternaria* **46**, 135–151, 10.4454/23dce671 (2023).

[73] Joly, P. F. *Carte Géomorphologique de reconnaissance de la presqu’île de Brögger (Spitsberg). 1:50 000.*Service de documentation et de cartographie géographiques du C.N.R.S. Institut du Géographie, https://esdac.jrc.ec.europa.eu/images/Eudasm/NO/PDF/nor_x17.pdf (1969).

[74] Geldard, J. *The production of a Quaternary geological map of Endalen, Svalbard, and assessment of Holocene geomorphic processes.* MSc thesis. University of Sheffield and University Centre in Svalbard, https://www.unis.no/wp-content/uploads/2021/05/UNIS_Annual_Report_2020_web.pdf (2019).

[75] Härtel, S. & Christiansen, H. H. Geomorphological and Cryological map of Adventdalen, Svalbard [dataset]. *PANGAEA*, https://doi.pangaea.de/10.1594/PANGAEA.833048 (2014).

[76] Rubensdotter, L., Romundset, A., Farnsworth, W. R. & Christiansen, H. H. *Landforms and sediments in Bjørndalen-Vestpynten, Svalbard. Quaternary geological map, scale 1:10 000*. Geological Survey of Norway (NGU), https://www.ngu.no/publikasjon/landskapsformer-og-losmasser-bjorndalen-vestpynten-svalbard-kvartaergeologisk-kart-110 (2015).

[77] Rubensdotter, L. & Jensen, M. *DynaCoast maps of Isfjord coastlines. 2014-2020, Scale 1:30,000.* SVALCOAST, https://svalcoast.com/maps/ (2026).

[78] Rouyet, L. *et al*. Seasonal InSAR Displacements Documenting the Active Layer Freeze and Thaw Progression in Central-Western Spitsbergen, Svalbard. *Remote Sensing* **13**, 2977, 10.3390/rs13152977 (2021).

[79] Lauknes, T. R., Zebker, H. A. & Larsen, Y. InSAR Deformation Time Series Using an L1 -Norm Small-Baseline Approach. *IEEE Transactions on Geoscience and Remote Sensing* **49**, 536–546, 10.1109/tgrs.2010.2051951 (2011).

[80] Rouyet, L., Eckerstorfer, M., Lauknes, T. R. & Riise, T. *Deformasjonskartlegging rundt Longyearbyen ved bruk av satellittbasert radarinterferometri* [Deformation mapping around Longyearbyen using satellite-based radar interferometry]. Norut Report 13/2017, 46 pp., https://www.miljovernfondet.no/wp-content/uploads/2020/02/17-59-terrengstabilitet-lyr.pdf (2018).

[81] Norwegian Polar Institute (Norsk Polarinstitutt, NPI). *Terrengmodell Svalbard (S0 Terrengmodell)* [Digital Elevation Model dataset], 10.21334/NPOLAR.2014.DCE53A47 (2014).

[82] Sessford, E. G., Bæverford, M. G. & Hormes, A. Terrestrial processes affecting unlithified coastal erosion disparities in central fjords of Svalbard. *Polar Research* **34**, 24122, 10.3402/polar.v34.24122 (2015).

[83] Strzelecki, M. C. *et al*. Cryo-conditioned rocky coast systems: A case study from Wilczekodden, Svalbard. *Science of the Total Environment* **607-608**, 443–453, 10.1016/j.scitotenv.2017.07.009 (2017). 28704670

[84] Planet Labs PBC. *Planet Application Program Interface: In Space for Life on Earth*, https://api.planet.com (2023).

[85] Norwegian Directorate for Cultural Heritage (Riksantikvaren). *Kulturminner - Lokaliteter, Enkeltminner og Sikringssoner*[Cultural Heritage Sites - Locations, Single Monuments and Protection Zones], https://kartkatalog.geonorge.no/metadata/kulturminner-lokaliteter-enkeltminner-og-sikringssoner/c72906a0-2bc2-41d7-bea2-c92d368e3c49 (2024).

[86] Longyearbyen Community Council (Longyearbyen Lokalstyre). *Bygning_polygon.shp* [unpublished dataset]. Obtained via personal email communication. Contact person: Jostein Børve Hernes. Email communication: 21 February 2025 (2025).

[87] Norwegian Polar Institute (Norsk Polarinstitutt, NPI). *NyAalesundomr_NorskPolarinstitutt_FKBC_Bygning_f.shp*; *NyAalesundomr_NorskPolarinstitutt_Tillegg_Nybygg.shp*; *NyAalesundomr_KingsBay_Kartverket_Brandallaguna_TMP.shp* [unpublished dataset]. Obtained via personal email communication. Contact person: Anders Skoglund. Email communication: 19 August 2025 (2025).

[88] Statistics Norway (Statistisk sentralbyrå). *Standard for bygningstype/Matrikkelen* [Standard for building types in the Norwegian Cadastre], https://www.ssb.no/klass/klassifikasjoner/31 (2000).

[89] Norwegian Mapping Authority (Kartverket). *BygningstypeKode BYGGTYP_NBR* [Building Type Code], https://objektkatalog.geonorge.no/Objekttype/Index/EAID_2F97667F_2BDF_4817_9B7E_821CF61212ED (2025).

[90] Kings Bay. *Plan description LAND-USE PLAN Ny-Ålesund 2024–2034*, https://kingsbay.no/wp-content/uploads/2024/05/Plan-description-land-use-plan-NyA-2024-2034_30.04.24.pdf (2024).

[91] Arctic Centre, University of Groningen. *The Netherlands Arctic Station*, https://www.arcticstation.nl (2025).

[92] Varnes, D. J. *Landslide hazard zonation: A review of principles and practice*. UNESCO, Paris, 63 pp. (1984).

[93] Fell, R. *et al*. Guidelines for landslide susceptibility, hazard and risk zoning for land use planning. *Engineering Geology* **102**, 85–98, 10.1016/j.enggeo.2008.03.022 (2008).

[94] Nicu, I. C. *et al*. Permafrost-related hazard, vulnerability and risk estimate for cultural heritage and modern buildings in Svalbard [dataset]. *Zenodo*, 10.5281/zenodo.18154592 (2026). PMC13658782 42251039

